
JOURNAL INFORMATION
==============================
NLM Title Abbreviation: Behav Neurol
Journal ID: Behav Neurol
NLM Journal ID: 8914585
Journal ID: BN

Behavioural Neurology

ISSN: 0953-4180
EISSN: 1875-8584
Publisher: Wiley

ARTICLE INFORMATION
==============================
PMCID: PMC5434322
PMID: 21422558
DOI: 10.3233/BEN-2010-0298
Article ID: 282950
Article version: 1
Subjects: Research Article

The Role of Posterior Parietal Cortex in Spatial Representation of Time: A TMS Study

Magnani Barbara 1 *
Oliveri Massimiliano 2 3
Mangano Giuseppa Renata 2
Frassinetti Francesca 1
1 Department of Psychology University of Bologna Bologna Italy
2 Department of Psychology University of Palermo Palermo Italy
3 Fondazione “Santa Lucia” IRCCS Rome Italy
*Barbara Magnani: barbara.magnani2@unibo.it
Print publication date: 2010
Electronic publication date: 2011 Mar 21
Volume: 23
Issue: 4
First page: 213
Last page: 215
Received 2011 Jan 31; Accepted 2011 Jan 31
Copyright: Copyright © 2010 Hindawi Publishing Corporation and the authors.
Copyright year: 2010
License: This is an open access article distributed under the Creative Commons Attribution License, which permits unrestricted use, distribution, and reproduction in any medium, provided the original work is properly cited.
License URL: https://creativecommons.org/licenses/by/3.0/

==============================
Behavioural Neurology 23 (2010) 213–215
DOI 10.3233/BEN-2010-0298
IOS Press

### The role of Posterior Parietal Cortex in spatial representation of time: A TMS study

Barbara Magnani[a,*], Massimiliano Oliveri[b,c], Giuseppa Renata Mangano[b] and Francesca Frassinetti[a]
[a]*Department of Psychology, University of Bologna, Bologna, Italy*
[b]*Department of Psychology, University of Palermo, Palermo, Italy*
[c]*Fondazione "Santa Lucia" IRCCS, Rome, Italy*

### 1. Introduction

The existence of a spatial representation of time, where temporal intervals are represented on a mental temporal line (MTL), oriented in ascending order from left to right, was demonstrated manipulating spatial attention by means of Prismatic Adaptation (PA). In young healthy subjects, prisms adaptation inducing a rightward shift of spatial-attention produced an overestimation of time intervals, whereas prisms adaptation inducing a leftward shift of spatial-attention produced an underestimation of time intervals [4]. The aim of the present study was to investigate the neural basis mediating the effects of PA on spatial time representation.

Posterior-Parietal-Cortex (PPC) is the best candidate to discharge this function. Indeed, neuropsychological and neurophysiological studies designate right-PPC as the site of space-time interaction [1,3,7]. Concerning the neural bases of PA procedures, left and right-PPC are involved in different phases of PA procedure [2,5, 6].

Here we investigated, by using TMS, the role of the Posterior-Parietal-Cortex (PPC) in spatial representation of time and in cerebral plasticity phenomena mediating prismatic adaptation effects on time processing. To this aim, healthy subjects were submitted to a temporal task before and after PA. TMS was applied on left or right PPC, before or after PA.

If the right (left) PPC is the key area leading the effects of PA on time, a reduced effect of PA on time is expected for TMS over right (left) PPC *both before and after PA*. If the right (left) PPC plays a role in PA procedure and not in mediating PA effects on time, the effect of TMS over right (left) PPC is expected for TMS applied *before and not after PA.*

### 2. Methods

A blue square (1cmx1cm) was presented on the center of the PC screen for a variable time interval (1600, 1800, 2000, 2200, 2400 ms) (encoding). After exposure to the blue square, a red square was presented and subjects were required to reproduce half the duration of the blue square (time bisection task) by pressing the space-bar on the keyboard.

Prismatic lenses inducing rightward shift of spatial attention were used (for details of PA procedure see [4]).

rTMS was applied over the parietal scalp sizes corresponding to P3 and P4 position of the 10–20 EEG system (for details of rTMS protocol see [7]).

Twenty-four right-handed healthy-subjects (1 men; range = 19 to 26 years; mean age = 21.71) participated in the study. Subjects were divided in four groups (six persons each), submitted to TMS on right-PPC, before (G1) and after-PA (G2), and on left-PPC, before (G3) and after-PA (G4). Subjects performed a

*Corresponding author: Department of Psychology, University of Bologna, Viale Berti Pichat, 5–40127 Bologna, Italy. Tel.: +39 051 209 1847; Fax: +39 051 243086; E-mail: barbara.magnani2@unibo.it.

ISSN 0953-4180/10/$27.50 © 2010 – IOS Press and the authors. All rights reserved

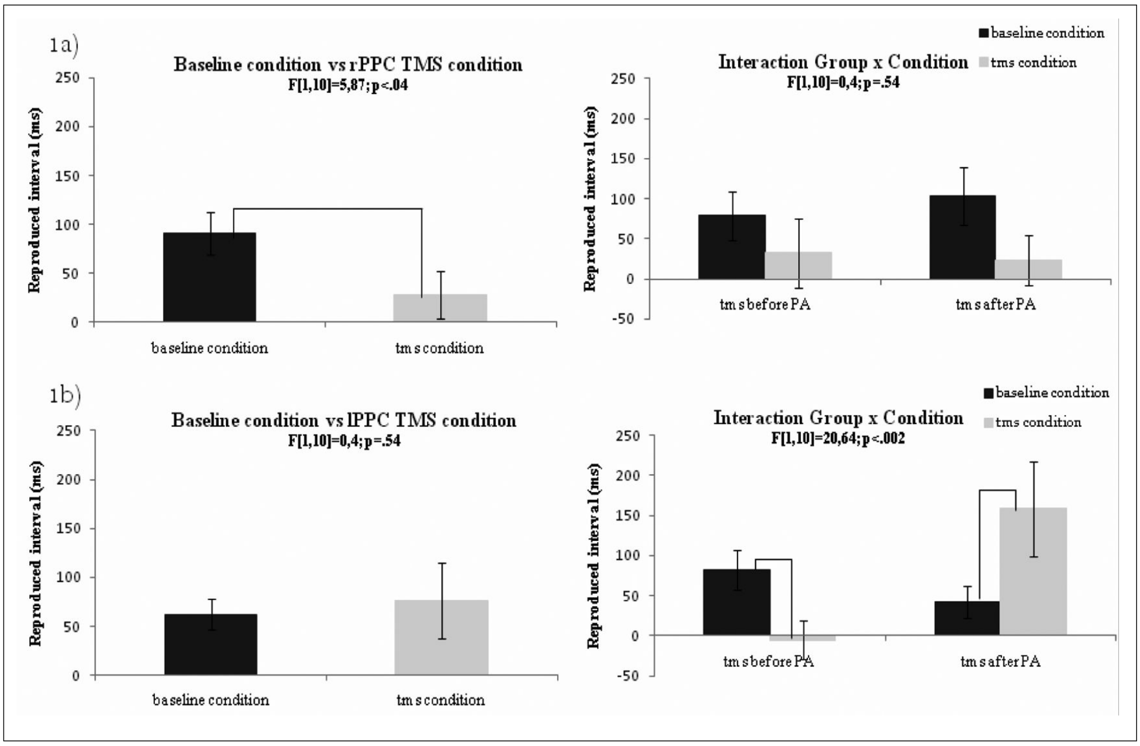

Fig. 1. Mean difference between reproduced time intervals before-PA minus after-PA, in baseline condition and in tms condition, for TMS over right-Posterior Parietal Cortex (r-PPC) (1a) and over left-Posterior Parietal Cortex (l-PPC) (1b) before-PA (tms before PA) and after-PA (tms after PA). Larger values indicate an overestimation.

*baseline condition* (B-condition) and a *TMS condition* (TMS-condition). In the baseline condition subjects were submitted to the time task before and after-PA. In the TMS-condition TMS was applied on right-PPC or on left-PPC, before and after-PA, according with the previously assigned Group (G1-G4).

### 3. Results and discussion

To verify the effects of PA on spatial time representation, subjects' performance before and after-PA was compared (T-test). Results showed that time intervals were overestimated after-PA respect to before-PA (977 ms vs 1052 ms, $p < 0.0001$).

To check whether *TMS on the right-PPC* interfered with the effects of PA on time, an ANOVA with Group as between-groups variable (G1 and G2) and Condition (B-condition vs TMS-condition) as within-groups variable was performed. Analysis was conducted on the mean difference between reproduced time intervals before-PA minus after-PA (ms), so that larger values indicate overestimation. Condition was significant [F(1,10) = 5,87; $p < 0.04$]: the overestimation found in B-condition was reduced in TMS-condition (91 ms vs 28 ms). Since Group and Group X Condition were not significant, TMS on right-PPC reduces the effect of PA on time regardless it is delivered before or after-PA (Fig. 1a).

A similar ANOVA was performed with G3 and G4 as between-groups variable, to verify whether *TMS on the left-PPC* interfered with the effects of PA on time. The interaction Group X Condition [F(1,10) = 20,64; $p < 0.002$] showed a reduction of the overestimation induced by PA when TMS was applied before-PA (G3) (82 ms vs $-6$ ms, $p < 0.05$), and an increment of the overestimation induced by PA when TMS was applied after-PA (G4) (42 ms vs 158 ms, $p < 0.02$) (Fig. 1b).

Finally the influence of TMS on PA procedure, was explored by using two separate ANOVAs (one for G1/G2, and one for G3/G4). Analysis were conducted on the mean of pointing displacement (degrees of visual angle) after prismatic lenses exposure (the so called after-effect). Group was considered as between-groups variable and Condition (B-condition vs TMS-condition) as within-groups variable. For right-PPC no significant effects were found. For left-PPC the interaction Group X Condition was significant [F(1,10)

$= 4{,}97$; $p < 0.05$]: after-effect was reduced in TMS-condition respect to B-condition for TMS applied before-PA (2,5° vs 3,6°, $p = 0.07$) but not after-PA (3,6° vs 3,5°, $p = 0.99$).

### 4. Conclusion

Results suggest that right-PPC plays the most important role in mediating the effect of PA on spatial representation of time, since a reduced effect of PA on time representation (a reduced overestimation) was obtained after TMS over right-PPC, independently from TMS was applied before or after PA.

Left-PPC interferes with PA procedures since the effects on time were evident only when TMS was applied before PA. Moreover, left-PPC acts inhibiting the effects of the right-PPC, actually its inhibition increased the effects of PA on time.

### References

[1] G. Basso, P. Nichelli, F. Frassinetti and G. di Pellegrino, Time perception in a neglected space, *Neuroreport* **7**(13) (1996), 2111–2114.

[2] H.L. Chapman, R. Eramudugolla, M. Gavrilescu, M.W. Strudwick, A. Loftus, R. Cunnington and J.B. Mattingley, Neural mechanisms underlying spatial realignment during adaptation to optical wedge prisms, *Neuropsychologia* **48**(9) (2010), 2595–2601.

[3] J. Danckert, S. Ferber, C. Pun, C. Broderick, C. Striemer, S. Rock and D. Stewart, Neglected time: impaired temporal perception of multiseconds intervals in unilateral neglect, *Journal of Cognitive Neuroscience* **19**(10) (2007), 1706–1720.

[4] F. Frassinetti, B. Magnani and M. Oliveri, Prismatic lenses shift time perception, *Psychological Science* **20** (2009), 949–954.

[5] J. Luauté, C. Michel, G. Rode, L. Pisella, S. Jacquin-Courtois, N. Costes, F. Cotton, D. le Bars, D. Boisson, P. Halligan and Y. Rossetti, Functional anatomy of the therapeutic effects of prism adaptation on left neglect, *Neurology* **66**(12) (2006), 1859–1867.

[6] J. Luauté, S. Schwartz, Y. Rossetti, M. Spiridon, G. Rode, D. Boisson and P. Vuilleumier, Dynamic changes in brain activity during prism adaptation, *Journal of Neuroscience* **29**(1) (2009), 169–178.

[7] M. Oliveri, G. Koch, S. Salerno, S. Torriero, E. Lo Gerfo and C. Caltagirone, Representation of time intervals in the right posterior parietal cortex: implication for a mental time line, *Neuroimage* **46**(4) (2009), 1173–1179.
